# Natural Course of *Chlamydia trachomatis* Bacterial Load in the Time Interval between Screening and Treatment in Anogenital Samples

**DOI:** 10.1371/journal.pone.0145693

**Published:** 2015-12-29

**Authors:** J. A. M. C. Dirks, G. A. F. S. van Liere, S. Bogers, N. H. T. M. Dukers-Muijrers, P. F. G. Wolffs, C. J. P. A. Hoebe

**Affiliations:** 1 Department of Medical Microbiology, Maastricht University Medical Center, School of Public Health and Primary Care, Maastricht, the Netherlands; 2 Department of Sexual Health, Infectious Diseases and Environmental Health, Public Health Service South Limburg, Geleen, the Netherlands; The University of Texas at San Antonio, UNITED STATES

## Abstract

**Introduction:**

Although *Chlamydia trachomatis* (CT) is the most common bacterial sexually transmitted infection worldwide, little is known about the natural course of the bacterial load during infection. We investigated the natural course of the bacterial load in the interval between screening and returning for treatment in genital and anorectal CT-infections.

**Materials & Methods:**

CT-positive patients, visiting our STI-clinic in the Netherlands from June 2011–January 2014, provided a second urogenital and/or anorectal sample when returning for treatment (diagnostic sample = T1; treatment sample = T2). Patient-record provided data about the days between samples and the date of last unsafe sex. Included patients were ≥18 years old, HIV-negative and did not report antibiotic use in the study-interval. CT load was quantified using qPCR. CT load was log-transformed, and a CT load difference (Δ-CT load) of >1 log was deemed clinically relevant. Chi-square test compared load category distributions over time (decrease/equal/increase), between sample types.

**Results:**

274 patients provided 296 paired samples. Majority of samples had a stable CT load in the interval T1-T2 (66.3%, 73.1% and 48.6% for vaginal swabs, urine and anorectal swabs resp. p = 0.07). Load decreased in 17–41% of patients, while ±10% of patients showed an increase in CT load. No association between Δ-CT load and the interval T1-T2 was observed. Large variations can be seen in CT load at T1 and over time.

**Discussion:**

The majority (±90%) of patients have a stable or decreasing CT load in the time interval between screening and returning for treatment. The number of days between sampling was not associated with change in CT load. In the first month after the last unsafe sex, only stable CT loads were seen. Our data seems to indicate that when most patients visit an STI-clinic, recommended 2 weeks after infection, the infection has already been established or is in its downward phase.

## Introduction

Although *Chlamydia trachomatis* (CT) is the most common bacterial sexually transmitted infection (STI) worldwide, with over 100 million people affected [1], little is known about the natural course of this infection. It is known that urogenital CT infections can have severe outcomes such as pelvic inflammatory disease and tubal scarring resulting in infertility and ectopic pregnancy in women [2]. However, spontaneous clearance of the bacteria has also been reported [3–7]. Too little is currently known about the natural course of a CT infection in humans to help predict the outcome, or even the duration of an infection in an individual patient. This may profoundly influence recommendations for control efforts such as STI screening frequency or time parameters for partner notification and treatment [3, 5].

Estimates of the duration of untreated CT infections, are highly variable by more than 40-fold, from 0.07 years to 2.99 years [8]. The number of patients that spontaneously clear their infection, ranges from 0–94% in studies [6, 9] depending on the time interval between tests. It is difficult to truly study the natural course of CT infections, as it is unethical to leave patients untreated after diagnosis. Moreover, nearly nothing is known about the natural course of the bacterial load during CT infections in humans.

The natural course of the bacterial load of *Chlamydia muridarum* in mouse models is more extensively studied. After CT administration, a sharp increase in CT load is seen, followed by a longer plateau phase, and eventually a decline in load until resolution, approximately 3–5 weeks after infection [10–12]. In humans, it is not unlikely that a similar load curve will be found, both in urogenital and anorectal samples [13]. However, all studies assessing CT load before treatment have determined load at a single time point during an infection [14–16].

We evaluated the bacterial load in two consecutive samples (one diagnostic sample and one sample before treatment) from CT-positive STI-clinic attendants to better understand the natural course of the urogenital and anorectal bacterial load over time.

## Materials & Methods

### Study population and procedures

From June 1st 2011 to January 2014 at the STI-clinic of Public Health Service South Limburg, the Netherlands, CT-positive patients of at least 18 years that attended one of five trained STI nurses partaking in the study, were asked to participate in this study by taking an extra self-collected vaginal swab for women and a first-void urine sample for men when returning for treatment. Some patients had been tested anorectally (i.e. all MSM and women based on their sexual history of receptive anorectal intercourse, and/or on reported anorectal symptoms) at the diagnostic visit and these participants also provided an extra self-collected anorectal swab when returning for treatment. For the purpose of this study, the first (diagnostic) sample that patients provided will from hereon be referred to as T1 and the second sample patients provided at the time of treatment as T2. Trained study nurses provided patients with a visual diagram and oral instructions about how to take separate self-collected vaginal and anorectal swabs. For the vaginal swab, the patient was instructed to insert the swab 2.5 cm into the vagina, rotate it for 5 to 10 seconds, and then place it in a capped tube to avoid potential contamination. This procedure was repeated in the anus for the anorectal swab. The rationale for using self-collected vaginal swabs for routine CT testing is that they have a similar or higher sensitivity compared to nurse-collected cervical swabs [17–19]. Specimens were screened for CT at the clinical microbiology laboratory at Maastricht University Medical Center (Maastricht, The Netherlands) using a commercial nucleic acid amplification test [polymerase chain reaction (PCR); Roche Cobas Amplicor until 2012 and later Roche Cobas 4800, Roche Diagnostics, Basel, Switzerland] as PCR has a higher sensitivity than culture based techniques [20].

**Fig 1:**
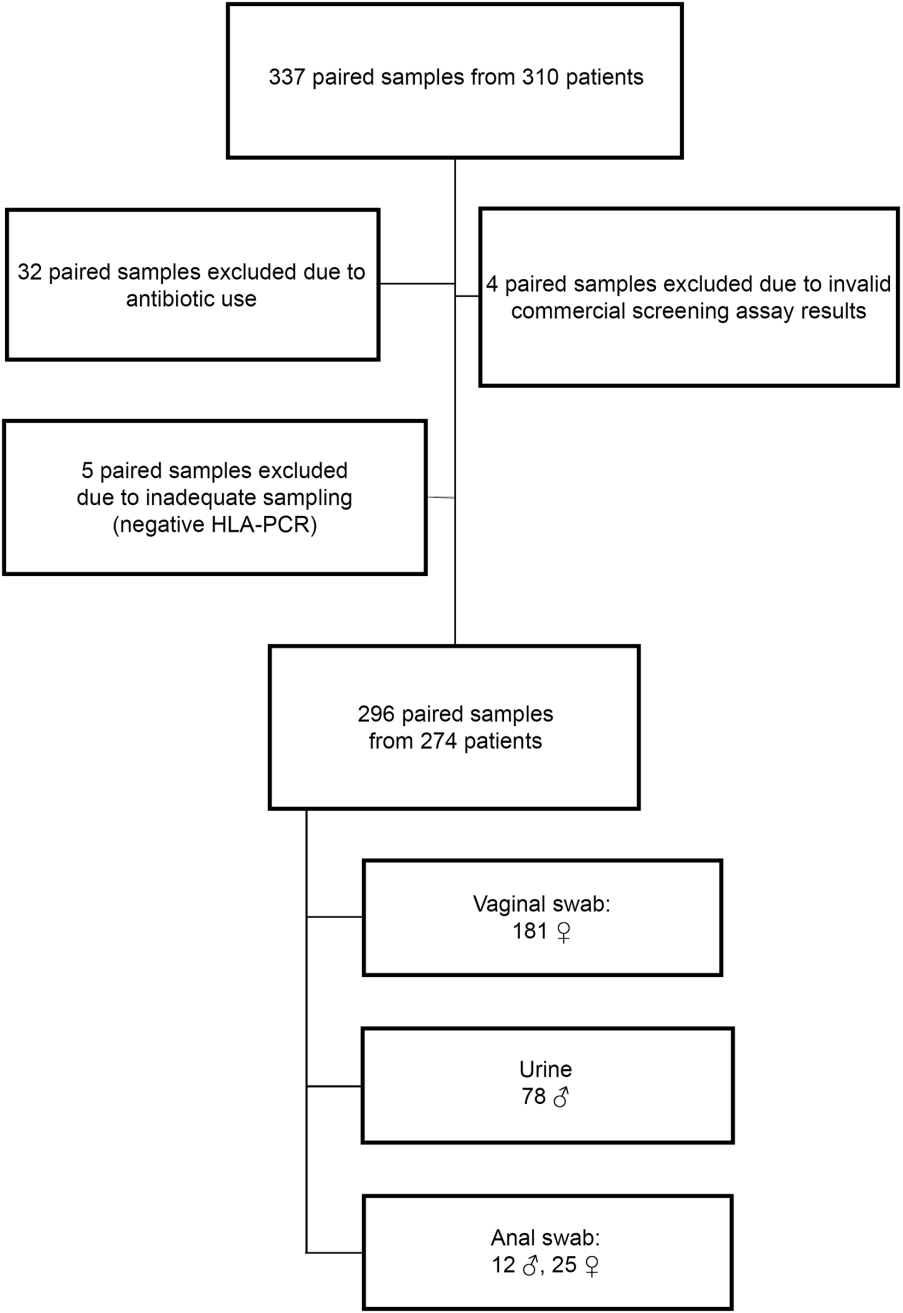
Flow chart of patients who met inclusion/exclusion criteria for the study population. 310 patients participated in the study, providing 337 paired samples. Forty-one paired samples did not meet inclusion criteria due to antibiotic use (n = 32), an invalid/inhibited commercial screening assay result (n = 4) or due to inadequate sampling as demonstrated by a negative *HLA*-PCR (n = 5). In total, 274 patients with 296 paired samples were included in this study (181 vaginal swabs, 78 urines and 37 anorectal swabs).

Patients empirically treated at the time of initial evaluation for CT-associated syndromes (e.g., urethritis or cervicitis) or other treatment indications (e.g., CT contact) were ineligible for inclusion in the study as the natural course of infection cannot be studied after treatment. Additional exclusion criteria for the current study were antibiotic use in the month before T1, or between T1 and T2, HIV-positivity and an invalid/inhibited CT-test result. This resulted in the exclusion of 41 paired samples from 36 patients (Fig 1).

A 30-day maximum time interval between T1 and T2 was allowed. Standard data registration at each consultation included sociodemographic information, antibiotic use, and for some patients also the date of the last unsafe sexual contact.

### Ethics statement

Written informed consent was obtained from all patients prior to participation in the study. This study, including the consent procedure, was approved by the Medical Ethics Committee of the Maastricht University Medical Center (METC azM/UM nr. 10-4-66; 15-6-2011).

### CT load quantification

When proven CT-positive at T1with the commercial screening assay, CT load quantification was performed for the T1 and T2 sample by an in-house qPCR as described by Dirks *et al*.[14]. In short, Taqman real-time PCR was used to quantify chlamydial *OmpA*-gene copies/ml and human leukocyte antigen (*HLA*)-gene copies/ml to ensure adequate sampling took place. Paired samples were excluded if no HLA could be detected in either sample (n = 5). Due to the lower sensitivity of the qPCR compared to the commercial screening assay, CT-positive samples with a CT load below the quantification limit were set to half the quantification limit[14].

Samples that were CT negative at T2 with the commercial screening assay, were included in this study as samples with 0 CT/ml. Every test is limited through a detection probability of samples with a low load, and we cannot rule out a very low CT load in these ‘CT-negative’ samples, despite duplicate testing.

### Load analyses over time

Load values were log transformed for analyses. Logarithmic converted load at T1 was subtracted from T2 to create a new variable ‘**Δ**-load’. A **Δ**-load difference of less than 1 log load (-1 to +1) is considered the cut-off value for a stable CT load over time when test-technical variability is taken into account. In short, a positive **Δ**-load value of >1 log indicates an increase in CT load, a load between +1 and -1 an equal CT load, and a negative value <-1 log a decrease in CT load.

The number of days between T1-T2 was used to construct a load curve in the short interval between T1 and T2. This natural load curve was then expanded by including the date of patients’ last unsafe sexual contact. The number of days since patients’ last (possible) exposure to CT was calculated by subtracting the date of patients’ last unsafe sexual contact from the consultation date (T1 and T2). This variable was then used to construct a natural load curve over time with two load measurements at distinct time points (T1 & T2), where T0 was the moment of the last sexual contact. Information about the date of the last unsafe sexual exposure was available for forty-six paired samples (15.5%), ranging from 3–565 days at T1 (median 80). As only 5 patients with anorectal swabs reported their last unsafe sexual contact, they were excluded from further analyses.

### Statistical analysis

Statistical analyses were stratified by sample type due to different load distributions per specimen (data not shown). Descriptive analyses included median and CT load range, the median and range of the number of days between T1-T2 and the median and range of days since last unsafe sexual contact. For the association between the CT load and time between T1-T2, Chi-square test was used to compare the distribution of load categories (decrease/equal/increase) between sample types. Delta CT load (Δ-CT load), 95% confidence intervals (95%CI) and p-values are shown. The mean period between sampling-moments was compared over sample types using one way ANOVA. Results were considered statistically significant at p≤0.05. All statistical tests were performed using IBM SPSS Statistics for Windows, version 22.0 (IBM Corp. Armonk, NY, USA).

## Results

### Characteristics of the study population

274 patients provided 296 paired samples (T1-sample and T2-sample treated as one pair of samples). Fig 1 provides a flowchart of the 296 included paired samples. Patient age ranged from 18–63 years [median 22].

### CT load distribution (in the time interval between T1-T2)

T1-samples had a CT load above the quantification limit of the qPCR in 85.8% (254/296), and T2-samples in 82.1% (243/296). At T2, 7.7% (n = 14) of vaginal swabs, 5.1% (n = 4) of urines and 10.8% (n = 4) of anorectal swabs were CT-negative according to the commercial screening assay (p = 0.36).

Table 1 shows the difference in CT load between samples, both absolute and in categories (increase/equal/decrease). Vaginal swabs had the largest range in CT load, varying from 0–2.2x10^8^ CT/ml, followed by anorectal swabs with a range of 0–1.4x10^6^ and the lowest range was detected in urines, from 0–1.3x10^5^ CT/ml.

**Table 1:** CT load distribution in absolute values (CT/ml) and 3 categories (decrease/equal/increase).

Sample type	N	Minimum	Maximum	T1 (median)	T2 (median)	Decrease % (n)	Equal % (n)	Increase % (n)
Vaginal swabs	181	0	2.2x10^8^	1.7x10^5^	1.5x10^5^	22.1% (40)	66.3% (120)	11.6% (21)
Urines	78	0	1.3x10^5^	2.5x10^2^	2.8x10^2^	16.7% (13)	73.1% (57)	10.3% (8)
Anorectal swabs	37	0	1.4x10^6^	3.9x10^3^	1.2x10^3^	40.5% (15)	48.6% (18)	10.8% (4)

The majority of samples had a stable CT load in the interval between T1-T2 (66.3%, 73.1% and 48.6% for vaginal swabs, urine and anorectal swabs respectively; p = 0.78). Increases in CT load were observed in up to 11.6% of samples and this proportion did not vary significantly between sample types (11.6%, 10.3%, 10.8% for vaginal swabs, urine and anorectal swabs respectively, p = 0.78). The proportion of samples in which the load decreased was the largest in anorectal swabs (40.5%), compared to urine (16.7%, p = 0.02) and vaginal swabs (22.1%, p = 0.059).

### CT load change related to the duration of the time interval between T1-T2

**Fig 2:**
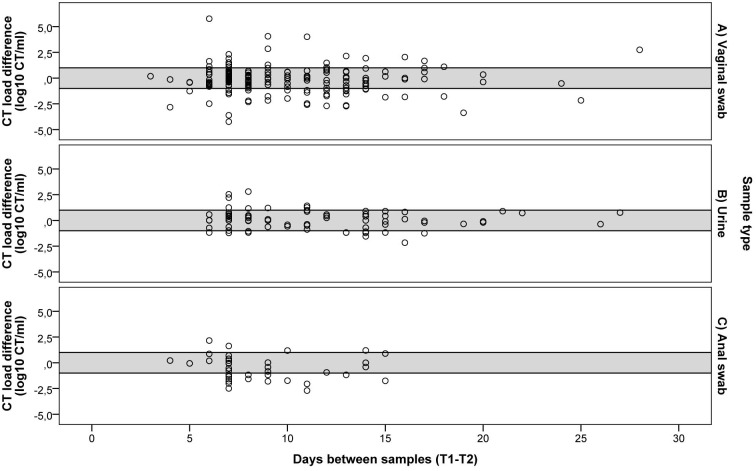
CT load in correlation to the time interval between T1-T2. CT load in A) vaginal swabs, B) urines and C) anorectal swabs. The grey area between lines (+1 to -1) indicates a stable Δ-CT load, the samples above the delineated area have an increase in CT load, and samples below the delineated area show a decrease in CT load in the time interval between T1-T2.

The median period between sampling moments was 8 days (range3-28 days) for vaginal swabs, 10 days (range 6–27 days) for urines and 7 (range 4–15 days) for anorectal swabs (p = 0.70). When the Δ-CT load is plotted in relation to the time interval between T1-T2, as can be seen in Fig 2, it becomes apparent that the majority of patients have a stable CT load over time, as the Δ-CT load lies most frequently in the area between -1 log and +1 log. Furthermore, a larger number of days between samples does not appear to increase the Δ-CT load, as no clear funnelling of the results is apparent. Statistically, no association between Δ-CT load and time interval between T1-T2 was observed for vaginal swabs (Δ-CT load -0.08, 95% CI -0.60–0.43, p = 0.75), for urine (Δ-CT load 0.24, 95% CI -0.28–0.77, p = 0.36) and for anorectal swabs (Δ-CT load -0.17, 95% CI -1.45–1.12, p = 0.80).

### CT load in the time since patients’ last unsafe sexual exposure

The load curve was constructed for forty-one paired samples (13.9%), of whom the last unsafe sexual contact was 3–565 days ago at T1 [median 90 days]. The median number of days since the last unsafe sex was 90 days (range 7–565) for vaginal swabs and 62 days (range 3–380) for urines.

**Fig 3:**
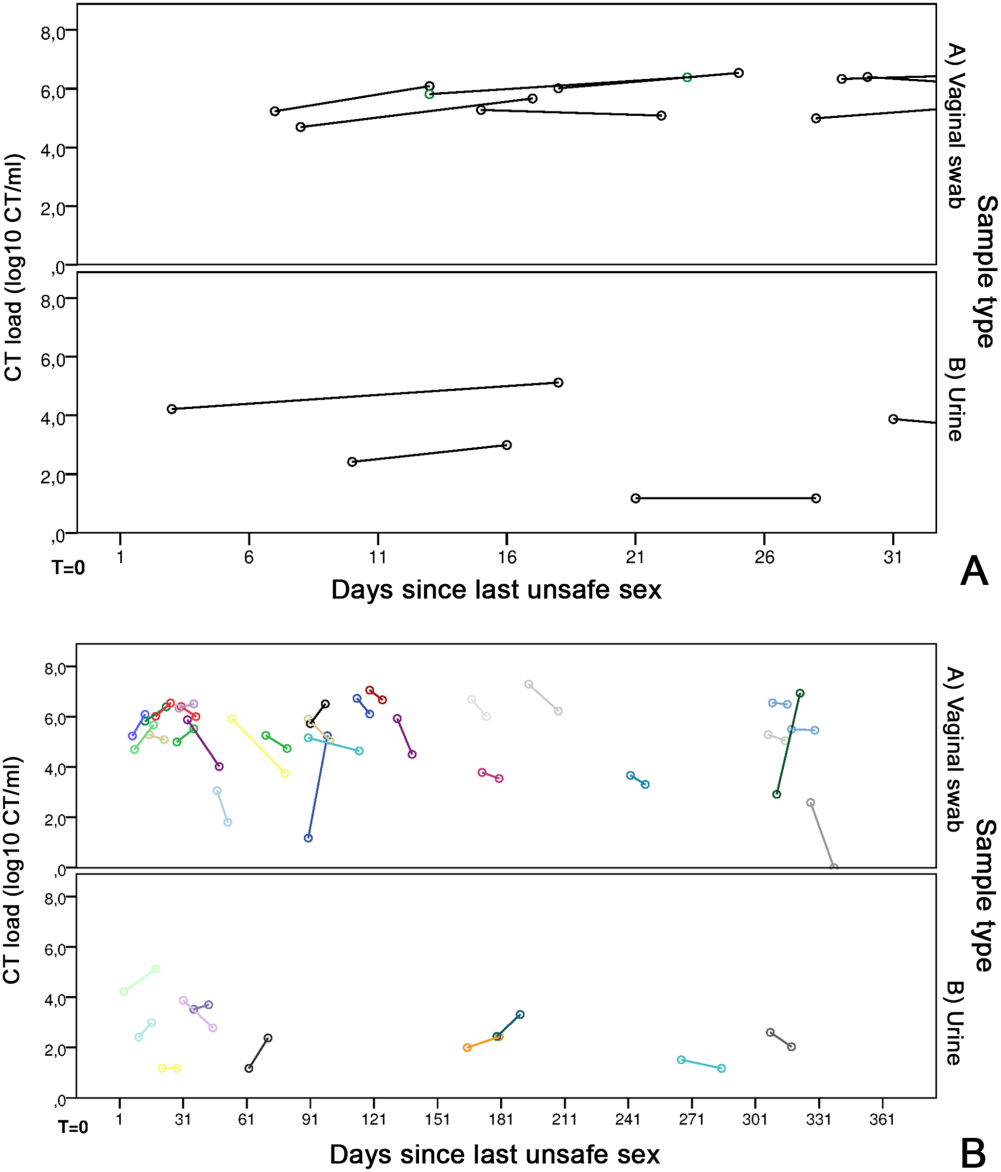
CT load in correlation to the time of patients’ last unsafe sexual exposure. (A) CT load in A) 29 vaginal swabs and B) 10 urines correlated to the last unsafe sexual contact of patients, for a maximum of 366 days. T = 0 is the moment of the last unsafe sex and each colour is a different patient. The first circle is the CT load at T1, and the second circle is the CT load at T2, where the length of the connecting line indicates the number of days between samples. (B) CT load in A) 8 vaginal swabs and B) 3 urines in the first month after the last unsafe sexual contact of patients. T = 0 is the moment of the last unsafe sex. The first circle is the CT load at T1, and the second circle is the CT load at T2, where the length of the connecting line indicates the number of days between samples.

The CT load curve for the first 366 days since the last unsafe sexual exposure (Fig 3A), shows large variations in each patient, in both in the CT load at T1, as well as the Δ-CT load over time. However, a general downward trend can be seen, with most patients exhibiting a decrease in CT load between T1-T2. The first 30 days after unsafe sexual exposure, which is likely the moment that most patients get tested for their CT infection are more clearly shown in Fig 3B. Here, only stable loads can be seen. Furthermore, none of the patients visiting the STI clinic in the two weeks after their last unsafe sexual contact had a ‘low’ CT load, i.e. a positive CT test in the commercial assay, but below the limit of quantification of our in-house PCR.

A decrease in CT load was first seen after one month (30 days) since the last unsafe sexual exposure. No patients showed an (sharp) increase in CT load in the first 2 months after the last unsafe sexual exposure.

Within 30 days after sexual exposure, CT load showed a median Δ-CT load of 0.53 log CT/ml (range -0.40–0.97), while the CT load after one month had a median Δ-CT load of -0.35 log CT/ml (range -2.58–4.06). Split by sample type, vaginal swabs had a median Δ-CT load of 0.53 log CT/ml (range -0.40–0.97) within 31 days, and -0.45 log CT/ml (range -2.58–4.06) after this time. Urines had a median Δ-CT load of 0.57 log CT/ml (range 0–0.90) within one month, and -0.45 log CT/ml (range -1.09–1.21) after one month.

## Discussion

We assessed the natural course of the CT load in the interval between screening and returning for treatment in patients visiting an STI-clinic patients. This is the first study to present quantitative data on the natural course of CT infections over time, in both urogenital infections and anorectal infections in men and women.

We demonstrated a stable CT load in the majority (47–73%) of patients in the time interval between screening and returning for treatment, which ranged in this study from 3–28 days. Depending on the sample type, load decreased in 17–41% of patients, while only ±10% of patients showed an increase in CT load. The number of days between T1-T2 did not correlate to the Δ-CT load, i.e. a longer time between sampling did not result in a larger difference in CT load. Furthermore, the time since last unsafe sexual exposure (to CT) was not clearly related to the course of CT load, with large variations over time.

Little is known about what determines CT load in humans [21], and even less is known about how it progresses during an infection. Prior studies (reviewed in [21]) have quantified load once during an infection, but never before were sequential time point analyses performed. It has become clear that the CT load can vary greatly, depending on the sample type, quantification methodology, age, immunological status, prior CT infections, and likely on the hormonal status of the patient [14, 22–24]. Furthermore, the moment of infection is always uncertain. To reconstruct the natural load-curve during CT infections, mice studies may be guiding. These studies showed a steady increase in bacterial load in the early hours after infection, after which the infection reaches a stable phase and after approximately a week a steady decline in the bacterial load sets in until resolution takes place [10, 11, 25, 26]. In our study, we demonstrated that the CT load is stable or decreasing in 90% of patients over time, both in urogenital and anorectal samples, consistent with the results found in animal studies. We correlated the number of days since patients’ last possible exposure to the CT load, but we could not demonstrate a similar sharp rise in the CT load in humans in the early moments after the last unsafe sexual exposure. We did see a minority of patients that experienced a sharp increase in CT load associated with the early stages of infection, congruent with murine studies, but we do not know their actual moment of infection. Unfortunately we did not have the data of last sexual exposure for all patients included in this study, while even this data would only provide a proxy of the actual moment of infection. Even after 3 days since their last unsafe sexual contact, patient load was sufficiently high to be detected through highly sensitive NAAT and in-house qPCR.

In this light, our results seem to indicate that the majority of patients visit the STI-clinic when the infection has already been established or is in its downward phase. This is surprising, as most current guidelines state that patients should get tested at least 2weeks after the last unsafe sexual practices based on CT’s incubation period of 1–3 weeks [27, 28]. Some recent guidelines incorporating current highly sensitive PCR-techniques are recommending immediate testing after CT exposure [4, 29], with the caution that a negative test must be repeated after 2 weeks. The data presented in this article provides new evidence that justifies the recent recommendations that the 2 week limit before tests might be let go.

Approximately 50% of CT-positives clear their urogenital infection within a year [3, 7]. In the interval between screening and treatment this number ranges from 9–44% in urogenital samples [3, 30, 31] and 18% (n = 11) in anorectal samples [13]. Contrastingly, we found few patients in whom spontaneous clearance occurred, only 8% (14/181) in vaginal swabs, 5% (4/78) in urines and 11% (4/37) in anorectal swabs, despite the use of tests with similar sensitivity and a similar test-interval [3, 32]. The low number of spontaneously cleared CT infections in our study can, most likely, be explained by the exclusion of patients who reported antibiotic use in the interval between T1 and T2 (9%). Anorectal load had a tendency to decrease more often than vaginal and urine load. A possible explanation is a more efficient immune response at the anorectal- than the urogenital site [33], but other factors may be at play.

A limitation of our study is the lack of data about prior CT infections in our patients, however, diagnosed prior infections are only a proxy of the total amount of previous CT infections and therefore may confound these analyses [31]. Another limitation of our study is the inability to perform serological analyses to provide insights into the natural course of CT infections (including possible persistent infections), as blood samples were only available for a minority of the patients included in this study.

It is very difficult to speculate on implications of these results on reinfection rates, progression of disease and associated pathology. Much is still unknown about the clinical implications of the bacterial load, and its correlation to pathology. A low CT load has been associated with subsequent clearance in pharyngeal positive patients [34]. In this study too (results not shown), patients with a low CT load cleared their infection most easily. Furthermore, it has been demonstrated that the CT load is lower in repeat infections [15, 16], and that naturally cleared infections protect from reinfection [31]. Furthermore, Russel *et al*. [35] demonstrated that seropositive patients had a lower cervical CT load than seronegative patients. However, it has also been shown that repeat CT infections increase the risk of CT-complications like tubal factor infertility [36]. Russel *et al*. [35] demonstrated that the CT load in the lower genital tract appears unrelated to the endometrial CT load, which is likely most important in developing CT sequelae. The patients with a high CT load are likely at high risk of infecting their sexual partner. It has been shown for other STI’s that a high infectious dose is more easily transmitted than a low dose [37–40]. Furthermore, these patients are more likely to have a continued immune stimulation beyond that of patients with a low CT load, and it may be that this increases their risk of complications. Although many hypotheses exist regarding the CT load and its effect on negative sequelae and re-infection rates of CT, too much is currently unknown to fully elucidate the processes at play.

In short, CT load is stable or decreasing in the vast majority of STI-clinic patients in the time interval between diagnosis and treatment. The time-interval between samples is unrelated to the change in load over time, requiring further research to clarify other factors at play. We found only stable CT loads within the first month after the las unsafe sex, which might reinforce recent guidelines that propose instant CT-testing after unsafe sexual practises.
